# Entrepreneurship education-infiltrated computer-aided instruction system for college Music Majors using convolutional neural network

**DOI:** 10.3389/fpsyg.2022.900195

**Published:** 2022-07-19

**Authors:** Hong Cao

**Affiliations:** School of Music and Dance, Hunan City University, Yiyang, China

**Keywords:** entrepreneurship education, aesthetic education perspective, convolutional neural network, auxiliary teaching system, Music Major

## Abstract

The purpose is to improve the teaching and learning efficiency of college Innovation and Entrepreneurship Education (IEE). Firstly, from the perspective of aesthetic education, this work designs the teacher and student sides of the Computer-aided Instruction (CAI) system. Secondly, the CAI model is implemented based on the weight sharing and local perception of the Convolutional Neural Network (CNN). Finally, the performance of the CNN-based CAI model is tested. Meanwhile, it analyses students’ IEE experience under the proposed CAI model through a case study of Music Majors from Xi’an Conservatory of Music. The experimental data show that the CNN-based CAI model can respond quickly and stably when users access different functional modules, such as webpage browsing. The proposed CAI model increases students’ entrepreneurial interest, skills, and knowledge by 55.62, 57.32, and 72.12%, respectively. Students’ entrepreneurial practice ability has been improved by over 50.00%; such an increase in entrepreneurial practice ability has also shown individual differences. Thus, the proposed Music Majors-oriented IEE-infiltrated CAI model based on CNN improves students’ entrepreneurial practice ability and reflects the positive experience of Music Majors on IEE. The finding provides references for the step-by-step identification of the CNN-based CAI model and has certain guiding significance for analyzing the effect of college IEE.

## Introduction

### Application background of computer technology in college innovative and entrepreneurship education

As China’s social progress accelerates, cultivating innovative, application-oriented, and entrepreneurial talents has been put on the domestic higher educational agenda (Wu and Wu, 2017). However, Innovation and Entrepreneurship Education (IEE) in Chinese Colleges and Universities (CAUs) is still in the exploratory stage and lacks effective and generalizable standards (Wu and Chen, 2021). For example, in the current college IEE, teachers assist classroom teaching using network platforms and teach the course as a career guidance handbook rather than a systematic and independent subject. Thus, much enhancement is needed in college IEE (Wu and Song, 2019). Fortunately, Computer-Based Education (CBE), first proposed in the United States in 1958, might provide a new outlook on college IEE (Hendikawati et al., 2019), especially in some well-known large-scale Computer-Aided Instruction (CAI) systems. The CBE saw its first breakthrough when the microcomputer was invented. It took a huge step forward when the CAI system came into being. Most recently, it has further integrated multimedia, network, and Artificial Intelligence (AI) technologies (Wu et al., 2021). Indeed, CBE and CAI are some major manifestations of the application of science in the educational field. The Chinese government has long been aware of the predominant role of Science and Technology (SandT) in social development. Thus, it has vigorously promoted online education to make up for traditional education, particularly IEE (Sinha and Bagarukayo, 2019).

### Application status of deep learning technology

On the other hand, music talents are special groups with exceptionally well-talented music skills and performance techniques. Therefore, infiltrating IEE into music education helps Music Majors fully display their talents. It gives students more freedom to think, judge, empathize, imagine, and express their feelings through music (Rogoza et al., 2018). Thus, IEE-infiltrated music education encourages creative and imaginative talents and helps them compose musical works and display music aesthetics to the public. Thereby, it popularizes music and promotes China’s music industry (Wu et al., 2019). In essence, IEE-infiltrated music education is a people-oriented aesthetic education. It coordinates the relationship between teaching, learning, and composing music well. It involves basic music knowledge and skills, the aesthetics theory, and the relationship between people and nature and society. Thus, it breaks free the spatial-temporal restrictions of teachers, students, and teaching, forming a Three-Dimensional (3D) and all-inclusive educational system (Guo et al., 2020). Most domestic CAUs have developed IEE and some entrepreneurship competition platforms through cooperation with enterprises. However, in practice, few students have been encouraged to participate, and less research is available (Zheng et al., 2018). By comparison, European and American countries have already introduced Maker Education for all college students’ daily courses. Many schools have set up special Maker courses and set up students’ Maker spaces to give students a platform to let their imagination land (Qian et al., 2018). In China, there was also sporadic IEE research. For example, a pilot project – online expatriate teachers–is a new form of “cloud friendship” to make up for Chinese expatriate teachers’ failure in overseas teaching due to the Corona Virus 2019 (COVID-19) outbreak (Chen, 2019). It was an online CAI system that provided a mobile learning platform (Wu et al., 2020). At the same time, many supportive technologies have been developed for the educational field. In 1962, the receptive field was coined and laid the foundation for developing the Neural Network (NN) and deep learning (DL) algorithms (Li et al., 2020). Subsequently, some researchers proposed neural cognition and weight sharing based on the receptive field (Shao et al., 2020). Then, these concepts were combined with the Backpropagation (BP) algorithm to construct the Convolutional Neural Network (CNN; Dzhezyan and Cecotti, 2021). All these technologies have shown great potential in the educational field. Thus, integrating state-of-the-art technology in IEE is an inevitable trend. Accordingly, this work introduces DL into the IEE-infiltrated music education.

### Writing framework

Currently, IEE in Chinese CAUs lacks an effective practice platform, and the instruction system is devoid of utility. This work aims to improve the effectiveness of college IEE and fill the research gap of a versatile and effective instruction system in the IEE development. Firstly, from the perspective of aesthetic education, the teacher side and student side of the CAI system are designed. Secondly, the CAI model is implemented based on CNN’s weight distribution and local perception. The innovation is to test the performance of the proposed CAI system on the CNN framework by investigating and analyzing the IEE experience of Music Majors from Xi’an Conservatory of Music. The logical structure is shown as follows. In Chapter 1, the background of CNN and CAI is introduced. Chapter 2 introduces the concepts of the CAI system and IEE. Then, in Chapter 3, the performance of the proposed CAI system is demonstrated. Chapter 4 analyses the data results and draws the research conclusions. This work can provide some references for designing and optimizing CAI systems through advanced technologies like DL. It also contributes to integrating IEE with other subjects to improve students’ Entrepreneurial Spirit (ES) and learning effect.

## Materials and methods

### Computer-aided instruction system from the perspective of aesthetic education

**FIGURE 1:**
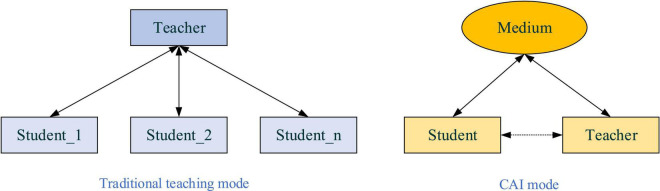
Traditional teaching mode and computer-aided instruction (CAI) mode.

The traditional college music education in China fails to consider the multicultural environment in curriculum design. It cannot arouse students’ music learning or composition initiative. Students learning interest in the music is lost. Besides, Music Majors are special students and rely more on practice than theoretical learning, which has not been fully reflected in the existing music education modes (Hosseini et al., 2019). Most often than not, students’ performance is mainly assessed by paper examination, without factoring in the multicultural elements and the weight of practical skills. Thus, those who do qualify mostly present a narrow knowledge base. Worse still, graduates lack the essence of music talents: creativity and the love of music as an art. Such being the case, it is urgent to expand music educational content, cultivate students’ creativity, and encourage their love for art through music aesthetics. To this end, some have introduced computer technology to implement the CAI system to improve the quality and efficiency of music education. At the same time, given the talents’ lack of creativity, the IEE can be infiltrated into music education as a novel attempt to improve the educational environment and teaching approach. Additionally, the music talents’ love for art can be fostered by aesthetic education. Thus, a fusion of different disciplines in music education can manage and coordinate the relationship between educators, students, and teaching and learning behaviors from a comprehensive and more scientific perspective (Hagiwara et al., 2020). Figure 1 shows the traditional teaching mode and CAI mode.

**FIGURE 2:**
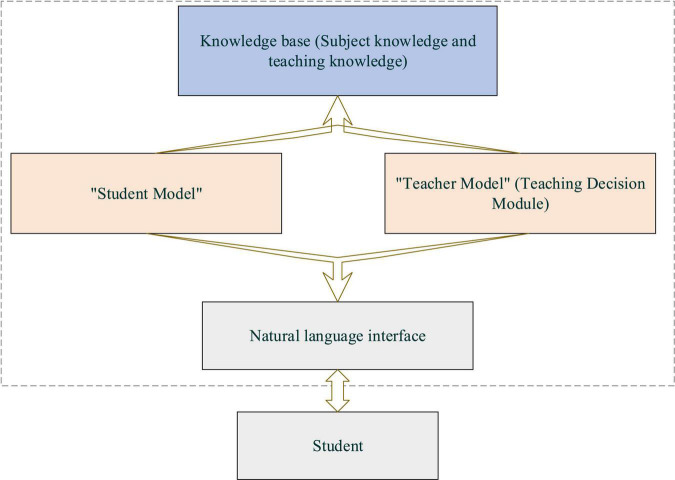
Basic structure of Institute for computer-assisted information (ICAI).

As shown in Figure 1, in the traditional teaching modes, teaching is mainly conferred through lectures or on-board notes, is straightforward, and involves no multimedia elements. In CAI mode, multimedia elements and computer and network technologies visualize classroom teaching, realize teacher-student interaction, and make learning more interesting (Yuan and Wu, 2020). At the same time, aside from imparting knowledge, teachers focus more on encouraging students’ participation in classroom activities and boosting their learning enthusiasm. Further, with the integration of Artificial Intelligent (AI) in education, Institute for Computer-Assisted Information (ICAI) analyses the characteristics and processes of students’ learning and thinking. ICAI aims to promote the intelligent development of the cognitive learning mode and teaching process (Zhang, 2021). Figure 2 is the basic structure of ICAI.

**FIGURE 3:**
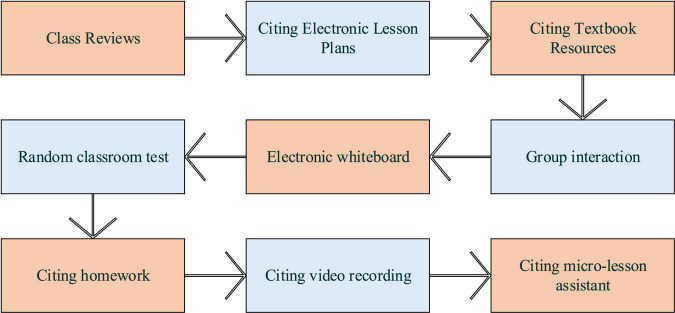
Teacher-side design process of CAI system.

In Figure 2, a natural language interface connects college students and teaching programs. Students’ and teachers’ behavior patterns might vary in different learning environments and instruction systems (Shogren et al., 2019). Thus, creating a pleasant learning environment with aesthetic pleasure can positively impact learning and teaching behaviors. Teachers should master various rules of formal beauty and enhance the consciousness and ability to beautify the teaching process. This has led to the necessity of aesthetic education that begins with formal beauty. Here aesthetic education uses the psychophysiological reaction law of humans to shape, sound, and color to strengthen college students’ aesthetic perception of the teaching process (Eryong and Li, 2021). Behaviorism learning theory holds that human thinking results from interaction with the external environment, namely “stimulation-response” or reinforcement. Environmental change and behavior reinforcement help create, design, shape, and change human behaviors. In particular, CAI should commend and encourage students’ ideal behavior and avoid negative reinforcement like punishment. Only positive reinforcement over negative reinforcement can expect a satisfactory teaching goal and learning effect (Muhajirah, 2020). Thus, behaviorism learning theory puts reinforcement at the core of teaching programs and believes in the promotion effect of a sound learning environment on students’ learning motivation. That is to say; the instruction system improvement should strengthen students’ learning experience in the CAI system and improve their aesthetic experience. Meanwhile, the CAI system is acted on by two different roles: teachers and students. Figure 3 is the teacher-side design process of the CAI system.

**FIGURE 4:**
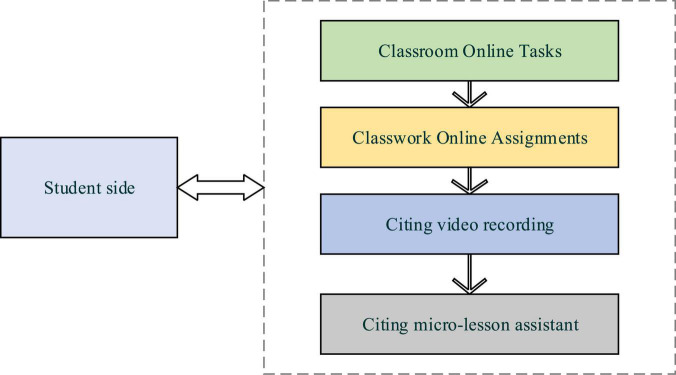
Student-side design process of the CAI system.

Figure 3 implies that the teacher side of the CAI system includes six processes: class review, citing electronic teaching plans, citing textbook resources, group interaction, electronic whiteboard, citing random classroom tests, citing homework, citing video recording, and citing micro-lesson assistants. Teachers use the class review to comment on students’ academic achievements and stimulate students’ learning. Teachers use electronic lesson plans to prepare lessons and apply them directly in class. The primary purpose of citing textbook resources is to insert textbook resource videos in the classroom. Teachers interact in groups, increase group interaction links, and make online comments in the teaching process. Meanwhile, the electronic whiteboard is used for teachers to write the teaching contents other than the electronic teaching plan. Teachers use random classroom tests to push the pre-written in-class test questions to students. The citing homework is where the teacher pushes the prepared homework to the students. Lastly, video records in class can be reviewed after class. Micro-lesson assistant realizes intelligent video editing during class. Figure 4 gives the student-side design flow of the CAI system.

According to Figure 4, the student side of the CAI system includes online classroom tasks, classroom online homework, citing video recording, and citing micro-lesson assistants. The system can improve students’ curriculum experience in learning and interactive teaching. Classroom online tasks can push teaching tasks from teachers to students, and students log in to the system to complete online tasks. Video recording is used for the video collection of sand painting platforms to watch the teacher’s teaching demonstration perfectly and simultaneously record the students’ practice process. Students mainly use the micro-lesson assistant to complete video editing and synthesis.

### Convolutional neural network theory and its application in instruction system

Neural network models simulate the structure and behavior of the biological neuron system for distributed and parallel information processing. It can process information by adjusting the weight between neurons. In particular, CNN, a feedforward Neural Network (NN) model with several convolution layers and pooling layers, can well lend to image processing (Zhang et al., 2021). Meanwhile, CNN has a deep structure and can complete convolution operations or the “filter operation” in image processing (Dhillon and Verma, 2020). For example, the convolution of the m*n convolution kernel on an original image *X* reads:


(1) $$z=w_{1}\,x_{1}+w_{2}\,x_{2}+\cdots +w_{m\,n}\,x_{m\,n}=w^{T}\,X$$

*w_1_*, *w_2_*, and *w*_*mn*_ are the weights of convolution kernel *W*. *x_1_*, *x_2_* and *x*_*mn*_ are the corresponding pixels on the original image. *z* is the value of the original image *X* after convolution. The convolution kernel convolutes and traverses the image using a certain-step sliding window. The sliding window will skip some pixels. Here, the convolution kernel covers 9 pixels in the original image each time. The output of an *F*-step convolution on an *n*-size original image reads:


(2) $$O=\frac{n-f}{s}+1$$

*O* indicates the size of the output image, and *s* denotes the convolution step. Convolution operations shrink and deform the image. The image edge is formed through one-pixel output, while the image center is formed through multiple overlapping convolution kernels. Thus, some edge pixels will be lost in convolution. The edge pixels in the center of the convolution kernel are extended to the false pixels outside the edge in the convolution kernel. Then, Eq. 3 calculates the size of the output image:


(3) $$O=\frac{n+2\,p-f}{s}+1$$

**FIGURE 5:**
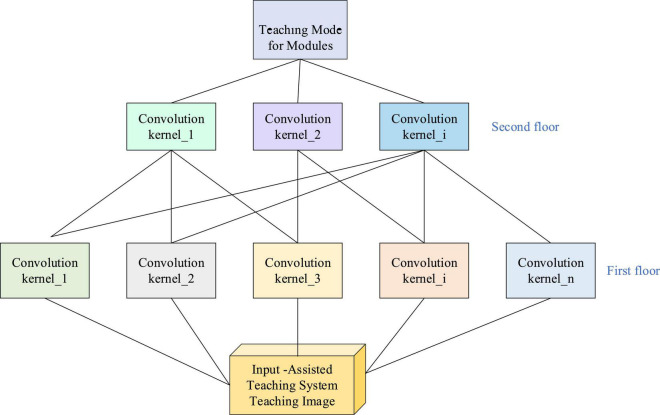
Convolutional neural network-based CAI model.

*p* denotes the filled pixel size, and then the image size of the original image is*n*+2*p*. Images’ spatial connection is also a local pixel connection: the closer the pixels, the stronger the correlation is. The correlation of distant pixels is weak. Therefore, each neuron does not need to perceive the global image. Global information can be obtained by perceiving local information and integrating it at a higher level. The structure of the visual system inspires the idea of a partial network connection in biology. Neurons in the visual cortex receive information locally (that is, these neurons only respond to stimuli in certain areas). Here, the CNN intelligent sensor identifies the CAI activities under different modes. Learning activity-oriented motion and speech recognition is to classify the accelerometer data sequence recorded by the special strap or smart computer and mobile phone into known and clearly defined motions. Figure 5 depicts the proposed CNN-based CAI model.

Figure 5 shows the pattern space hierarchy of the CNN-based CAI system. The first convolution layer will learn smaller local patterns. The second convolution layer will learn larger patterns composed of the features of the first layer. Iteratively, the next deeper layer will learn larger patterns of the features of the previous layer. The higher the number of levels, the more comprehensive learning features are. After learning, the response of different convolution kernels to different characteristics changes. Finally, the step-by-step implementation module is obtained for the CAI model. The CNN architecture is applied to the CAI model. Based on the main functions and theories of the CAI, the technical support and theoretical characteristics associated with DL are explored. The theory of DL is refined according to the actual teaching needs, which has practical reference value for improving the IEE of Music Majors.

### College Music Majors-oriented innovation and entrepreneurship education

College IEE improves students’ quality through the relevant curriculum system, such as the spirit of independence, innovation, adventure, as well as ES. Meanwhile, IEE reshapes students’ employment concepts and entrepreneurial beliefs (Aparicio et al., 2019). The IEE curriculum is comprehensive and practice-oriented, with complex and diverse assessment methods. So far, IEE in China lags far behind in higher education, especially for some art majors that need substantial amounts of practice, such as the Music Major. Music Majors can carry out practical simulations using the CAI model with an excellent experience. Thus, the CAI model helps construct a better IEE platform. Surely, the IEE-infiltrated music education will cultivate Music Major’s ES and innovative spirit, promoting economic development. This claim has also been confirmed by many scholars who have found the positive role of Music Majors in national economic development. For instance, Schediwy et al. (2018) conducted quantitative analysis and research on the attitude of Music Majors toward IEE. The research results showed that the values such as enthusiasm for music and the need for autonomy were not contradictory to the perceived needs of IEE related to professional work. McGee et al. (2021) studied the IEE framework of Music Majors. They promoted students’ self-guidance and guidance through adaptive curriculum planning. The results suggested that the program design could use the principles of social constructivism to provide novel IEE courses. Paulsen et al. (2021) discussed the enterprise innovation of art majors. The relationship between innovation, entrepreneurship and economic growth was investigated. The research had important reference values for the IEE reform of art majors. Table 1 lists the characteristics of IEE for Music Majors under the CAI system.

**TABLE 1:** Characteristics of college Music Majors-oriented innovation and entrepreneurship education (IEE).

Characteristic	Research object	Concrete implementation
Subjectivity	Music Majors	Students’ cognition and experience of entrepreneurship
Exploratory	IEE teaching process	Achieve the goal of college Music Majors-oriented IEE
Activity	IEE teaching activities	Teaching efficiency of IEE
Life style	IEE practice link	Examination of college Music Majors-oriented IEE

Analyzing the characteristics of college Music Majors-oriented IEE reveals that Music Majors’ cognition and entrepreneurship experience are subjective. The teaching process is exploratory. The efficiency of teaching activities needs to be improved, and the practice link needs to be re-examined.

### Questionnaire survey and research

Through the step-by-step implementation of the CAI system from the perspective of CNN and aesthetic education, 108 Music Majors in a college are randomly selected for a questionnaire survey (QS). Then, the invalid QSs, such as missed selection and multiple selections, are picked out and eliminated, with 98 valid ones reaching a 90.7% recovery rate. In order to verify the data reliability, Statistical Packages for Social Science (SPSS) and Cronbach’s α coefficient are used to analyze the reliability and validity of the QS. The data results show that the correlation coefficients of each dimension are higher than 0.7, indicating a good structural validity. The reliability of the QS is also analyzed. Lastly, the formal QS in the study is prepared according to the standard. The measured Cronbach’s α is between 0.75 and 0.90, which significantly conforms to the reliability range. The structure of the QS is given in Table 2:

**TABLE 2:** Designed IEE QS.

Thank you for taking part in the survey in your busy schedule. Please choose the answer truthfully.
1. Gender A. Male B. Female
2. Grade A. Freshman B. Sophomore C. Junior D. Senior
3. Are you an only child? A. Yes B. No
4. Have you ever been a student cadre? A. Yes B. No
5. Do you know about college students’ Entrepreneurship? A. Yes B. No
6. What is your career preference? A. Civil servant B. To go into an enterprise C. Seek further education D. Self-employed
7. Have you ever had the idea of starting a business while you are a student? A. Never B. Have C. Somewhat interested D. Very interested
8. What model of IEE have you received? A. Optional course B. Compulsory course C. Entrepreneurship lecture D. Entrepreneurship competition
9. What do you think are the elements of successful entrepreneurship? A. Personal capability B. Social relations C. Financial support D. Entrepreneurship policy of the college

Here is the specific experimental environment:64-bit Windows10 operating system; an Intel (R) Core (TM) i7-8700K 3.7 GHz Central Processing Unit (CPU); a 16GB Random Access Memory (RAM); and the NVIDIA GeForce GTX 1080Ti 8GB Graphics Processing Unit (GPU). Through the comparative analysis before and after using the proposed CNN-based CAI system, this work analyses the practical ability of Music Majors’ IEE. The CLPsyh2017 ReachOut dataset is also selected to train and test the CNN. The dataset includes 1,188 pieces of test data and 400 pieces of training data. The test and training data are sorted out to train and test the model, respectively.

## Results and discussion

### Performance test of proposed convolutional neural network-based computer-aided instruction model

**FIGURE 6:**
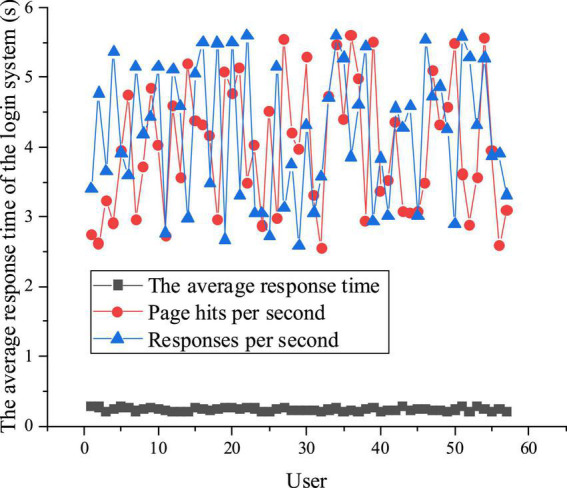
Performance test and analysis of the proposed CNN-based CAI model.

This section verifies the proposed CNN-based CAI model’s different module performance. The model training iteration *n* = 500. First, the open CAI system interface is closed, and users log in synchronously. When concurrent user number = 57, the performance of the proposed CNN-based CAI system is analyzed and tested. Figure 6 plots the performance test and analysis of the proposed CNN-based CAI model.

Figure 6 shows that the average system response time for recording and viewing the IEE scripts and logging in is 0.19–0.28 s. The proposed CNN-based CAI model can respond to 2.67 to 5.65 clicks/second for user web browsing, which can meet the user’s practical needs of 2.38 to 5.67 clicks/second. Thus, the proposed CAI model has a quick and stable response time for different modules.

### Analysis of the influence of the computer-aided instruction system on Music Majors’ innovation and entrepreneurship education

**FIGURE 7:**
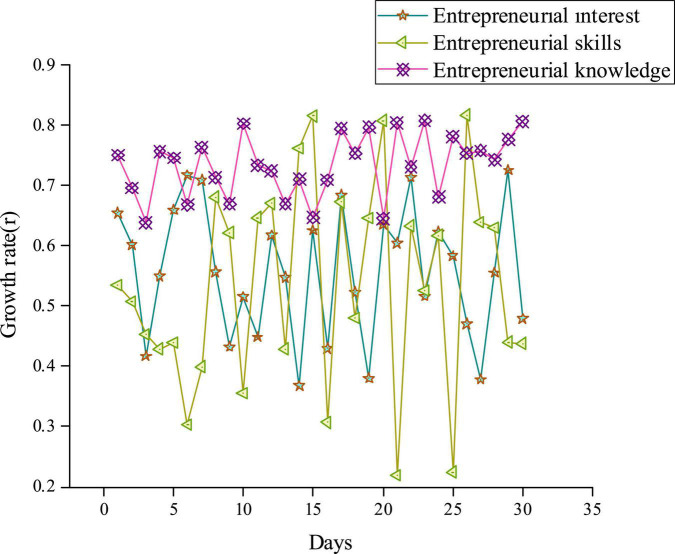
Analysis of the growth coefficients of Music Majors-oriented innovation and entrepreneurship education (IEE) after introducing CAI.

Subsequently, a QS is conducted on 108 Xi’an Conservatory of Music students. The proposed CNN-based CAI model is introduced in college IEE. The growth coefficient is used to study the changes in Music Majors’ entrepreneurial interest, entrepreneurial skills, and entrepreneurial knowledge within 1 month. Figure 7 analyses the results of the Music Majors-oriented IEE’s growth coefficient after introducing the proposed CNN-based CAI.

As in Figure 7, the proposed Music Majors-oriented IEE-infiltrated CAI model based on CNN has increased students’ entrepreneurial interest by 36.80% at least and 71.15% at best, with an average increase of 55.62%. Simultaneously, entrepreneurial skills have increased by 23.92% at least and 80.46% at best, with an average increase of 57.32%. Entrepreneurial knowledge has increased by 64.87% at least and 80.60% at best, with an average increase of 72.12%. Thus, the average increase in entrepreneurship knowledge is within the scope of practical consideration. Music Majors’ entrepreneurial interest and entrepreneurial skills have increased by half. The proposed Music Majors-oriented IEE-infiltrated CAI model can effectively improve Music Majors’ entrepreneurial interest, entrepreneurial skills, and knowledge. It provides references for applying the CNN and CAI systems in college IEE.

### Analysis of the practical ability of the proposed Music Majors-oriented innovation and entrepreneurship education-infiltrated computer-aided instruction model

**FIGURE 8:**
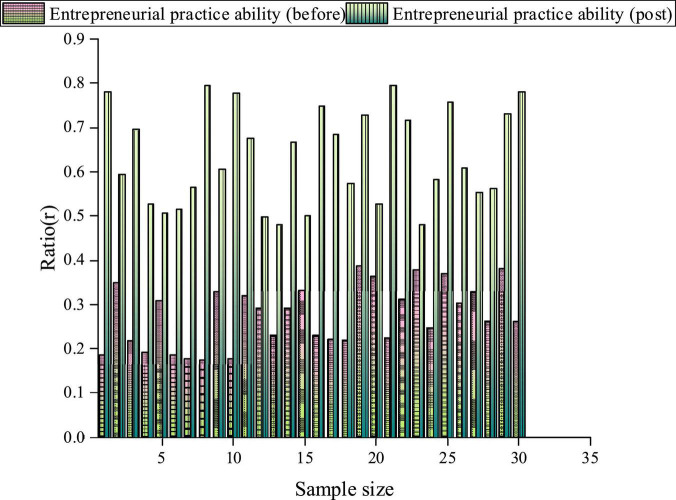
The practical abilities of Music Major college students.

Figure 8 compares the Music Majors’ abilities before and after introducing the proposed Music Majors-oriented IEE-infiltrated CAI model.

Figure 8 shows that Music Majors’ average score of practical entrepreneurial ability (pre-test) is 0.28 and 0.60 after introducing the proposed CAI model. Moreover, students have individual differences in each dimension after introducing the proposed CAI model. Such individual differences help confirm the research results’ reasonability. Specifically, students’ entrepreneurial practical ability has been improved by more than 50.00%. Thus, the proposed Music Majors-oriented IEE-infiltrated CAI model improves students’ entrepreneurial practical ability and reflects the positive effect of college IEE.

## Conclusion

In social progress and economic development, innovative and entrepreneurial talents have always been the main driving force. This work proposes a college Music Majors-oriented IEE-infiltrated CAI model based on CNN from the perspective of aesthetic education. It chooses Music Majors from Xi’an Conservatory of Music using the proposed CAI model to study their increase in practical abilities. The results show that with 57 concurrent users, the average response time of the proposed CAI model is between 0.19 and 0.28 s. The proposed CAI model can respond to 2.67–5.65 clicks/second for user web browsing, which can meet the user’s practical needs of 2.38–5.67 clicks/second. Thus, the proposed CAI model has a quick and stable response time for different modules. Music Majors’ entrepreneurial interest, skills, and knowledge have increased by 55.62, 57.32, and 72.12%, respectively, after introducing the proposed CAI model. Music Majors’ average practical entrepreneurial ability before and after introducing the proposed CAI model is 0.28 and 0.60, respectively. Therefore, the proposed Music Majors-oriented IEE-infiltrated CAI model based on CNN can improve Music Majors’ learning experience for IEE, entrepreneurial interest, skills, knowledge, and practice abilities. The proposed CAI model can provide some experimental reference for researching the influencing factors of college IEE. Last but not least, there are still some deficiencies. The QS is issued from the perspective of Music Majors, and the experience analysis of CAI and IEE is not comprehensive. In future research, it is prospected to expand the major and numbers of student samples and analyze the experience of college IEE from multiple perspectives. The aim is to provide some theoretical support for the development of CNN and college IEE.

## Data availability statement

The raw data supporting the conclusions of this article will be made available by the authors, without undue reservation.

## Ethics statement

The studies involving human participants were reviewed and approved by the Hunan City University Ethics Committee. The patients/participants provided their written informed consent to participate in this study. Written informed consent was obtained from the individual(s) for the publication of any potentially identifiable images or data included in this article.

## Author contributions

The author confirms being the sole contributor of this work and has approved it for publication.

## Conflict of interest

The author declares that the research was conducted in the absence of any commercial or financial relationships that could be construed as a potential conflict of interest.

## Publisher’s note

All claims expressed in this article are solely those of the authors and do not necessarily represent those of their affiliated organizations, or those of the publisher, the editors and the reviewers. Any product that may be evaluated in this article, or claim that may be made by its manufacturer, is not guaranteed or endorsed by the publisher.
